# Metabolome and transcriptome analyses reveal chlorophyll and anthocyanin metabolism pathway associated with cucumber fruit skin color

**DOI:** 10.1186/s12870-020-02597-9

**Published:** 2020-08-24

**Authors:** Min Wang, Lin Chen, Zhaojun Liang, Xiaoming He, Wenrui Liu, Biao Jiang, Jinqiang Yan, Piaoyun Sun, Zhenqiang Cao, Qingwu Peng, Yu’e Lin

**Affiliations:** 1 Vegetable Research Institute, Guangdong Academy of Agricultural Sciences, Guangzhou, 510640 China; 2 Guangdong Key Laboratory for New Technology Research of Vegetables, Guangzhou, 510640 China

**Keywords:** *Cucumis sativus* L., Metabolome, RNA-Seq, Chlorophyll, Anthocyanin

## Abstract

**Background:**

Fruit skin color play important role in commercial value of cucumber, which is mainly determined by the content and composition of chlorophyll and anthocyanins. Therefore, understanding the related genes and metabolomics involved in composition of fruit skin color is essential for cucumber quality and commodity value.

**Results:**

The results showed that chlorophyll a, chlorophyll b and carotenoid content in fruit skin were higher in Lv (dark green skin) than Bai (light green skin) on fruit skin. Cytological observation showed more chloroplast existed in fruit skin cells of Lv. A total of 162 significantly different metabolites were found between the fruit skin of the two genotypes by metabolome analysis, including 40 flavones, 9 flavanones, 8 flavonols, 6 anthocyanins, and other compounds. Crucial anthocyanins and flavonols for fruit skin color, were detected significantly decreased in fruit skin of Bai compared with Lv. By RNA-seq assay, 4516 differentially expressed genes (DEGs) were identified between two cultivars. Further analyses suggested that low expression level of chlorophyll biosynthetic genes, such as *chlM*, *por* and *NOL* caused less chlorophylls or chloroplast in fruit skin of Bai. Meanwhile, a predicted regulatory network of anthocyanin biosynthesis was established to illustrate involving many DEGs, especially *4CL*, *CHS* and *UFGT*.

**Conclusions:**

This study uncovered significant differences between two cucumber genotypes with different fruit color using metabolome and RNA-seq analysis. We lay a foundation to understand molecular regulation mechanism on formation of cucumber skin color, by exploring valuable genes, which is helpful for cucumber breeding and improvement on fruit skin color.

## Background

Fruit skin color is an essential trait with commercial values, mainly determined by content and composition of anthocyanins and chlorophyll [1, 2]. Chlorophyll provides green pigmentation and comprises with chlorophyll a and chlorophyll b molecules. Chlorophyll metabolism can be classified into three major steps: chlorophyll synthesis, chlorophyll cycle and chlorophyll degradation. A series of important enzymes were involved in chlorophyll metabolism, such as glutamyl-tRNA reductase (HemA), porphobilinogen synthase (HemB), magnesium chelatase subunit H (chlH), magnesium-protoporphyrin O-methyltransferase (chlM), protochlorophyllide reductase (por), chlorophyll b reductase (NOL) [3, 4]. Most fruit skin was caused by chlorophyll metabolism, which exhibit green color during the fruit early development, whereas the predominant colorations of yellow, orange and red show in the post stage [5–8].

Anthocyanins, the most prominent pigment influencing fruit color, were catalyzed by complex enzymes from phenylpropanoid and flavonoid biosynthetic pathways. A wide range of constructive genes were involved in the anthocyanin biosynthesis, such as phenylalanine ammonia lyase (*PAL*), 4-coumarate: coenzyme a ligase (*4CL*), chalcone synthase (*CHS*) and anthocyanidin synthase (*ANS*) [9–11]. Among them, *PAL* is an essential factor during the anthocyanin synthesis [12]. Flavonoid secondary metabolites are synthesized by a branched pathway of flavonols and anthocyanins synthesis. Previous study reported that various flavonoids exert crucial roles in protecting against UV-light and phytopathogens, development of male fertility, and transport of auxin [13]. Enzymes involved in anthocyanins and flavonoid synthesis are multi-enzyme complex [14], and pigments tend to accumulate in vacuole (anthocyanins and proanthocyanidins) or cell wall (phlobaphenes) [15].

Cucumber fruit skin color has great effect on commodity sale and varietal improvement. Previous studies concerning cucumber fruit skin color mainly focus on inheritance and gene primary mapping, such as white fruit skin gene (*w*), dark green fruit skin gene (*DG*), green fruit skin gene (*dg*), yellow green fruit skin gene (*yg*), and dull fruit skin light green fruit skin gene [16, 17]. The *w* was rapidly mapped to a 33.0-kb region by two SNP-based markers, ASPCR39262 and ASPCR39229 [18]. However, the molecular mechanism and pigment metabolism of fruit skin color in cucumber is unclear.

The combination of different omics helps us deeply understand several crucial genes involved in plant growth, development, and responses to different stresses [19, 20]. For instance, combined transcriptomic and metabolomics profiling offered some cues in explaining plant phenotype [21–23]. Through comparative transcriptomic analysis, reports showed that several novel genes functions were involved in the flavonoid [24] and other biochemical pathways [25]. In addition, metabolome efficiently analyzed genes roles involved in metabolic pathway and provided essential information on genes exploring [21]. The comparative omics has been successfully applied in fruits to clarify the relationship between different secondary metabolites and expressed genes [23]. However, until now, reports on regulation mechanism of cucumber fruit skin color by transcriptomic and metabolomics analysis still lack.

The aim of our study was to excavate the genes involved in development of cucumber fruit skin color using conjoint analysis. Two high-inbred cucumber genotypes, ‘Lv’ with dark green skin and ‘Bai’ with light green skin from South China type cucumber variety were applied. Comparison results showed that much more content of anthocyanins, flavone, and flavonols in the fruit skin of Lv compared with Bai. In addition, we detected that the key structural genes, transcription factors and other regulators during chlorophyll and anthocyanins biosynthetic pathways. We offered crucial information on fruit skin color and its complex effect on cucumber fruit quality.

## Results

### Phenotype analysis of lv and Bai

**Fig. 1:**
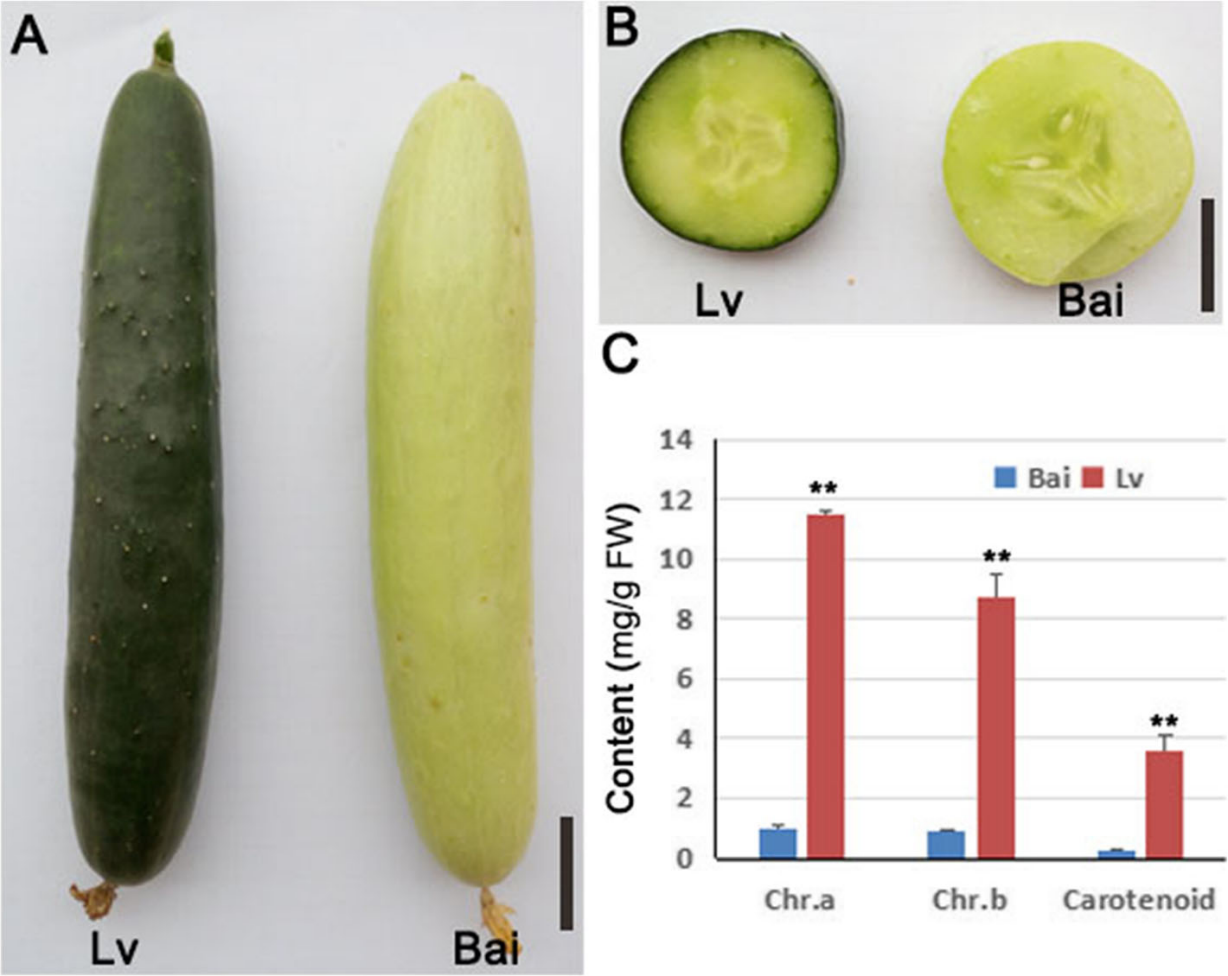
Phenotype of Bai and Lv about chlorophyll in fruit skins. **a** Fruit external characteristic of Lv and Bai. **b** Crosscutting observation of fruit from Lv and Bai. **c** Measurement of chlorophyll and carotenoid content of fruit skins from Lv and Bai. Scar bar in (**a**) 3 cm, (**b**) 2 cm. Data is presented as the mean ± standard deviation (*n* = 9). *0.01 ≤ *P* ≤ 0.05, **P ≤ 0.01, Student’s *t* test

Obvious differences were found between Lv and Bai in the young fruit skin color, the fruit skin color of Lv is dark green but Bai is light green (Fig. 1a, b). The content of chlorophyll a and chlorophyll b were 0.99 mg/g and 0.90 mg/g in Bai, respectively, which were significantly lower than the Lv (Fig. 1c). The result of carotenoid is consists with chlorophyll a and chlorophyll b, the carotenoid content was higher in Lv than Bai (Fig. 1c). These results indicating more pigments accumulated in Lv fruit skin.

**Fig. 2:**
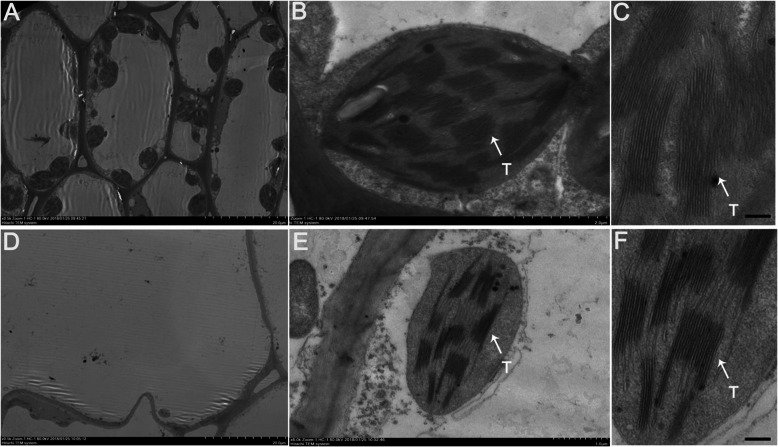
Transmission electron microscopy observation of Bai and Lv fruit skins. **a**-**c** Transmission electron microscopic photos of cells from Lv. **d**-**f** Transmission electron microscopic photos of cells from Bai. “T” in the figure represents thylakoid. Scar bar in (**a**), (**c**), (**d**) and (**f**) 20 μm, (**b**) 2 μm, (**e**) 1.0 μm

The above results indicated that more pigments accumulated in Lv fruit skin, which prompted us to further determine whether difference of chloroplasts in Lv and Bai cell. Through transmission electron microscopy (TEM) assay, we found that less chloroplast existed in Bai cells than Lv (Fig. 2a-c), and the number of thylakoid in a chloroplast of Bai (Fig. 2d-f) was less than Lv, these result was consistent with quantitative analysis of chlorophyll a and chlorophyll b.

**Fig. 3:**
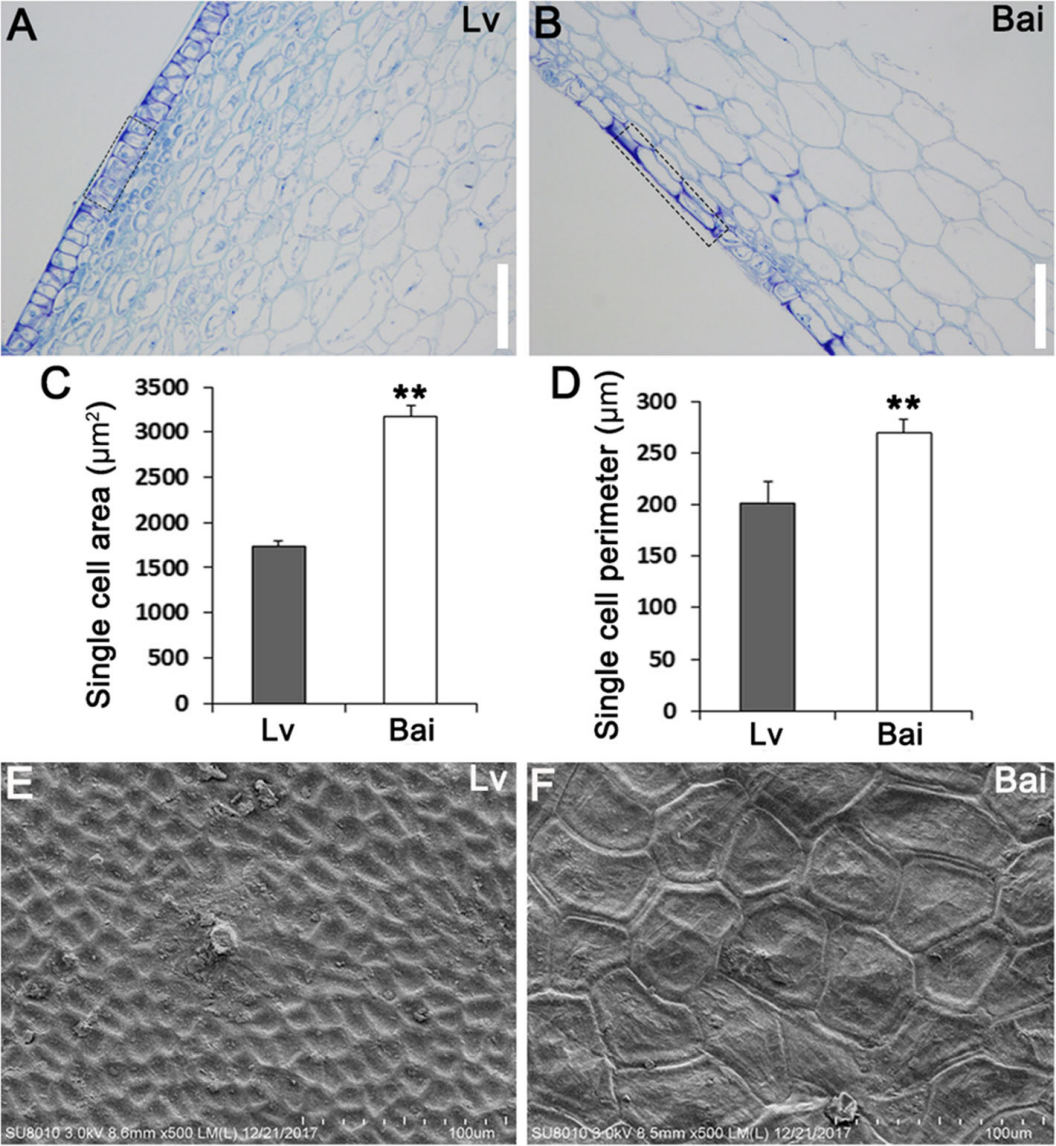
Epidermal cells from Bai showed larger single cell area and perimeter. **a**, **b** Observation of paraffin section of fruit skins from Lv (**a**) and Bai (**b**). **c** Single cell area of epidermal cells from Lv and Bai fruit skins. **d** Single cell perimeter of epidermal cells from Lv and Bai fruit skin. **e**, **f** SEM observation of fruit skin from Lv (**e**) and Bai (**f**). Scar bar in (**a**, **b**): 150 μm. Data is presented as the mean ± standard deviation (*n* = 9). *0.01 ≤ *P* ≤ 0.05, **P ≤ 0.01, Student’s *t* test

The paraffin section assay was carried out to observe arrangement of skin epidermal cells. The results showed that epidermal cells in Lv were more closely arranged than Bai (Fig. 3a, b). The single cell area and single cell perimeter of Bai were both lager than Lv (Fig. 3c, d). In addition, the surface cells on the Lv fruit skin were smaller than Bai in a same field of view by scanning electron microscope (SEM) assay (Fig. 3e, f, S1).

### Metabolite identification

**Fig. 4:**
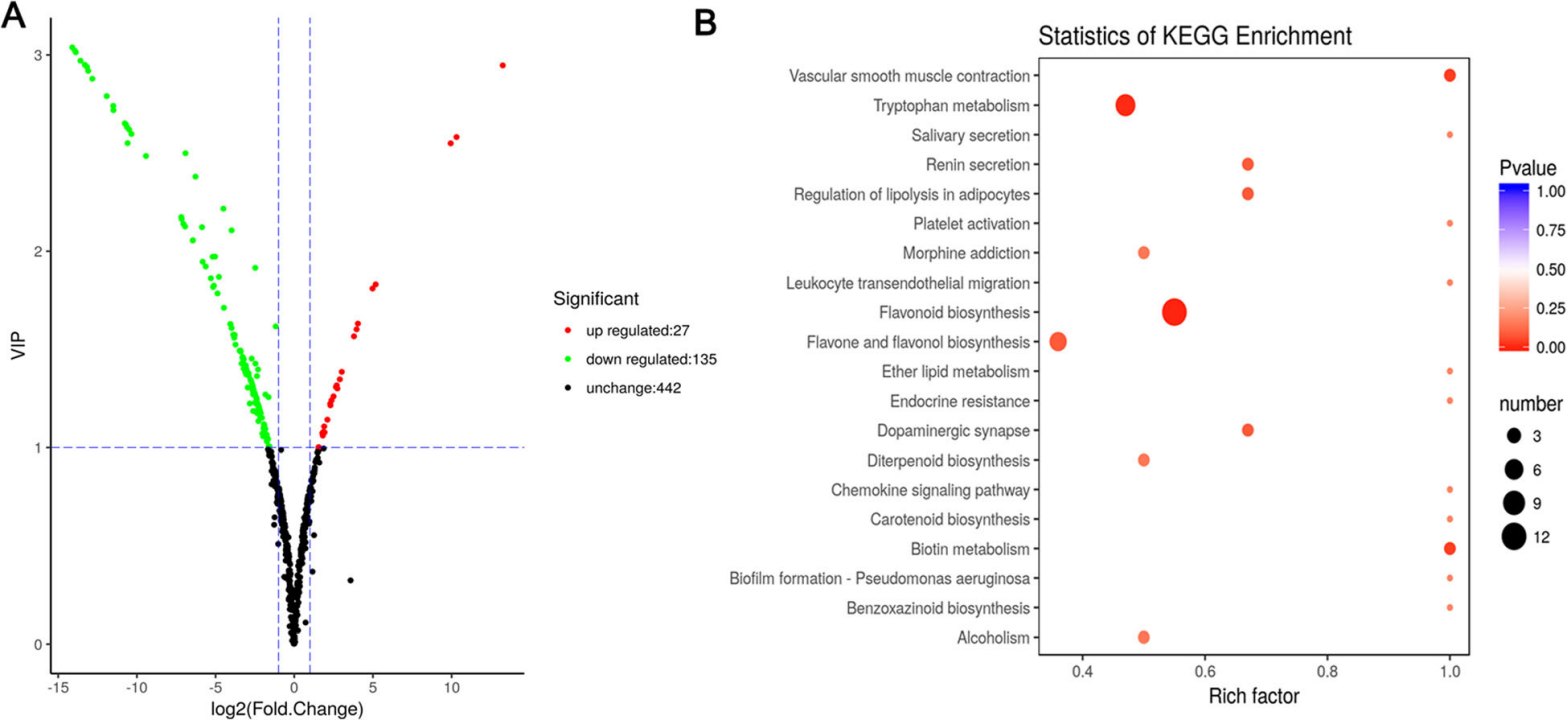
Comparison and KEGG analysis of different metabolites in fruit skin between Lv and Bai. **a** Different metabolites in fruit skins of Lv and Bai. Red, green and black correspond to up-regulated, down-regulated, and unchanged content of metabolites, respectively. **b** KEGG enrichment of annotated metabolites from Lv and Bai. The y-axis indicates the KEGG pathway and the x-axis indicates the enrichment factor

In order to excavate metabolites during the process of cucumber fruit development (Fig. 1), a metabolome program was performed in this study. Combing detection of total ions current (TIC) and multiple reactions monitoring (MRM) profiles, we finally identified 162 significant metabolites (135 up- regulated and 27 down-regulated) between Lv and Bai samples (Fig. 4a), including: 40 flavones, 9 flavanones, 7 flavonols, 6 anthocyanins, and other compounds (Table S1). The representative metabolites, especially anthocyanins, flavones, and flavonols were listed in Table 1.

**Table 1:** Differentially identified metabolites in the skin of Lv and Bai fruit

Class	Compounds	Lv	Bai	VIP	Fold_Change
Catechin derivatives	Protocatechuic acid O-glucoside	1.06E+ 06	1.73E+ 05	1.27	0.1633103
	L-Epicatechin	1.07E+ 04	7.16E+ 04	1.30	6.675995
	(+)-Gallocatechin (GC)	5.16E+ 03	3.84E+ 04	1.35	7.4370562
	Catechin	1.64E+ 04	5.91E+ 05	1.83	36.056911
Anthocyanins	Rosinidin O-hexoside	4.78E+ 04	1.30E+ 04	1.10	0.271777
	Cyanidin O-acetylhexoside	5.92E+ 03	9.69E+ 02	1.19	0.1636466
	Malvidin 3-O-glucoside (Oenin)	3.01E+ 04	3.56E+ 03	1.42	0.1182927
	Malvidin 3,5-diglucoside (Malvin)	2.53E+ 04	1.60E+ 03	2.11	0.0632632
	Peonidin O-hexoside	1.42E+ 04	9.00E+ 00	2.63	0.0006323
	Peonidin	2.59E+ 04	9.00E+ 00	2.72	0.0003479
	Selgin 5-O-hexoside	7.44E+ 04	1.97E+ 04	1.11	0.2653519
	Chrysoeriol	1.97E+ 04	3.84E+ 03	1.18	0.1949239
	Chrysoeriol 7-O-hexoside	5.67E+ 05	1.21E+ 05	1.20	0.2132785
	Chrysoeriol 5-O-hexoside	1.97E+ 06	4.10E+ 05	1.21	0.2084746
	Baicalein (5,6,7-Trihydroxyflavone)	5.73E+ 04	1.06E+ 04	1.26	0.1845751
	Chrysoeriol O-hexosyl-O-pentoside	1.16E+ 04	3.27E+ 03	1.27	0.2815805
	Tricin O-sinapoylhexoside	2.52E+ 05	3.83E+ 04	1.30	0.152053
	Luteolin 7-O-glucoside (Cynaroside)	1.05E+ 05	1.35E+ 04	1.31	0.1286574
	Tricin 7-O-feruloylhexoside	7.58E+ 04	8.38E+ 03	1.42	0.1105055
	Chrysoeriol O-malonylhexoside	1.15E+ 06	1.23E+ 05	1.45	0.1066667
	Acacetin O-acetyl hexoside	7.16E+ 05	6.70E+ 04	1.50	0.093622
	Butin	6.69E+ 04	2.04E+ 03	1.97	0.0305229
	Tricetin O-malonylhexoside	2.68E+ 06	2.03E+ 04	2.14	0.0075776
	Chrysoeriol O-hexosyl-O-rutinoside	1.74E+ 06	1.21E+ 04	2.17	0.006912
	Tricin O-sinapic acid	4.03E+ 04	1.78E+ 03	2.22	0.0441522
	Chrysoeriol O-hexosyl-O-hexosyl-O-Glucuronic acid	4.33E+ 04	5.59E+ 02	2.38	0.0129176
	Luteolin O-sinapoylhexoside	1.29E+ 04	9.00E+ 00	2.62	0.0006959
	Tricetin	1.10E+ 05	9.00E+ 00	2.97	8.16E-05
	Chrysoeriol O-sinapoylhexoside	1.41E+ 05	9.00E+ 00	3.02	6.37E-05
	Tricin O-glucuronic acid	1.25E+ 04	4.52E+ 04	1.08	3.6246993
Flavone	C-hexosyl-chrysoeriol O-hexoside	2.27E+ 04	6.84E+ 03	1.03	0.3007331
	Isovitexin	3.47E+ 04	8.54E+ 03	1.07	0.2462056
	8-C-hexosyl-apigenin O-feruloylhexoside	5.69E+ 04	1.57E+ 04	1.10	0.2754982
	8-C-hexosyl-hesperetin O-hexoside	3.27E+ 05	6.46E+ 04	1.23	0.1972505
	Apigenin 6-C-hexosyl-8-C-hexosyl-O-hexoside	5.01E+ 05	8.47E+ 04	1.28	0.1690619
	C-hexosyl-apigenin O-p-coumaroylhexoside	1.48E+ 04	2.68E+ 03	1.43	0.1806517
	Naringenin C-hexoside	1.55E+ 04	2.77E+ 03	1.92	0.1792672
	Chrysoeriol 6-C-hexoside 8-C-hexoside-O-hexoside	1.03E+ 07	1.82E+ 05	1.95	0.0177137
	6-C-hexosyl chrysoeriol O-hexoside	1.36E+ 06	1.56E+ 04	2.05	0.0114481
	Chrysoeriol 8-C-hexoside	1.61E+ 06	1.85E+ 04	2.06	0.0115145
	6-C-hexosyl-chrysoeriol O-feruloylhexoside	5.80E+ 05	9.97E+ 03	2.12	0.017199
	8-C-hexosyl chrysoeriol O-hexoside	1.57E+ 06	1.28E+ 04	2.13	0.0081529
	6-C-hexosyl-apigenin O-feruloylhexoside	2.82E+ 06	1.98E+ 04	2.16	0.0070178
	6-C-hexosyl-apigenin O-sinapoylhexoside	9.64E+ 04	7.99E+ 02	2.50	0.0082947
	di-C,C-hexosyl-apigenin	1.17E+ 04	9.00E+ 00	2.60	0.0007681
	8-C-hexosyl-chrysoeriol O-feruloylhexoside	3.48E+ 04	9.00E+ 00	2.79	0.0002589
	C-hexosyl-chrysoeriol O-sinapoylhexoside	6.55E+ 04	9.00E+ 00	2.88	0.0001374
Flavanone	Eriodictyol O-malonylhexoside	2.87E+ 04	7.40E+ 03	1.10	0.2579559
	Xanthohumol	2.39E+ 04	4.60E+ 03	1.25	0.1920613
	Naringenin 7-O-glucoside (Prunin)	1.16E+ 05	1.04E+ 04	1.49	0.0900834
	Naringenin	7.52E+ 04	2.73E+ 03	1.87	0.0362384
	Hesperetin	7.10E+ 04	1.95E+ 03	1.97	0.027507
	7-O-Methyleriodictyol	1.57E+ 04	9.00E+ 00	2.65	0.0005732
	Homoeriodictyol	8.32E+ 04	9.00E+ 00	2.94	0.0001081
	Naringenin chalcone	9.12E+ 04	9.00E+ 00	2.95	9.87E-05
	Naringenin O-malonylhexoside	1.58E+ 05	9.00E+ 00	3.04	5.68E-05
Flavonol	Quercetin 7-O-malonylhexosyl-hexoside	1.55E+ 05	2.49E+ 04	1.30	0.1612069
	Kaempferol 3-O-rhamnoside (Kaempferin)	1.13E+ 05	1.70E+ 04	1.34	0.1502941
	Kaempferol 3-O-rutinoside (Nicotiflorin)	2.21E+ 05	2.28E+ 04	1.46	0.1031627
	Kaempferol 3-O-robinobioside (Biorobin)	2.22E+ 05	1.41E+ 04	1.61	0.0632684
	Kaempferide	1.39E+ 04	9.00E+ 00	2.55	0.0006487
	Kaempferol-3-O-robinoside-7-O-rhamnoside (Robinin)	1.50E+ 04	9.00E+ 00	2.65	0.0006
	Fustin	9.00E+ 00	8.84E+ 03	2.55	981.85185

### Functional analysis of metabolites

Six rosinidin O-hexoside, cyanidin O-acetylhexoside, malvidin 3-O-glucoside, malvidin 3, 5- diglucoside, peonidin O-hexoside, and peonidin were identified and all these anthocyanins were significantly decreased in Bai fruit skin compared with Lv. In Bai, peonidin and cyanidin O-malonylhexoside were decreased with 0.00035- and 0.16-fold increments in contrast to Lv, indicating that lower content of anthocyanin partly caused slight hue of Bai (Table 1). Most flavonols were found with 0.006- to 0.16- fold augment in Bai except fustin, while content of fustin was prominently increased 981.85-fold in Bai compared with Lv. Flavones were detected to be the maximum number of metabolites among metabolites with the significant content changes between two cucumber genotypes. Among these, chrysoeriol O-hexosyl-O-rutinoside, and tricetin O-malonylhexoside, luteolin O-sinapoylhexoside demonstrated significantly higher content in Lv, while only tricin O-glucuronic acid was 3.62-fold increase in Bai (Table 1). In addition, KEGG (Kyoto Encyclopedia of Genes and Genomes) analysis demonstrated that different metabolites were mostly enriched in flavonoid biosynthesis and tryptophan metabolism, indicating flavonoid influenced fruit skin color development to some extent (Fig. 4b).

### Identification of differently expressed genes (DEGs) by transcriptome

**Fig. 5:**
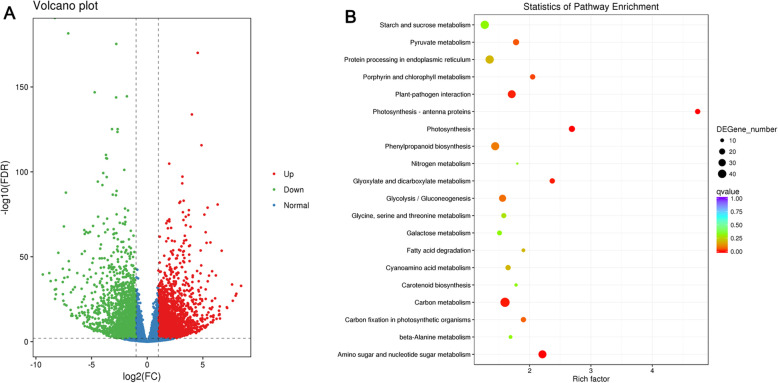
Comparison and KEGG analysis of DEGs in fruit skin between Lv and Bai. **a** Analysis of DEGs in fruit skin of Lv and Bai. Red, green and blue correspond to up-regulated, down-regulated, and normal content of metabolites, respectively. **b** Histogram of GO terms assigned to DEGs in fruit skin of Lv and Bai. All GO terms are grouped into three ontologies: green for biological process, orange for cellular component, and purple for molecular function

Total RNA from cucumber fruit skin were used for construction of cDNA libraries. After removing adaptor-containing raw reads and low-quality reads, the total number of clean reads was about 24 million for Lv and Bai (Table S2). These clean reads were subsequently mapped to cucumber 9930 genome (Huang et al., 2009). Approximately 90% clean reads were mapped to the reference cucumber genome, with more than 98% uniquely mapped (Table S2). The correlation coefficients in gene expression level from three biological replicates of each line were more than 0.84 (Fig. S2A), and principal component analysis (PCA) showed that biological replications clustered together (Fig. S2 B). The correlation coefficients and PCA suggested that expression patterns have similarity between replicate samples (Fig. S2). In total, 4516 DEGs with 2417 up-regulated and 2099 down-regulated genes were identified in Lv vs Bai.(Fig. 5a; Table S3). Combing transcriptome analysis, 205 DEGs belonged to 44 families encoding transcription factors (TFs), including 87 and 118 DEGs expressed down-regulation and up-regulation in Bai compared with Lv, respectively (Fig. S3). The AP2/ERF, bHLH, MYB, NAC and WRKY families were the top five TF in DEGs (Fig. S3). A total of 15 genes were selected to confirm RNA-seq data by using qRT-PCR, including 9 and 6 genes were selected from down-regulatin and up-regulation, respectively. The qRT-PCR results were consistent with RNA-seq data (Fig. S4). In addition, *Csa3G904140* was detected different expressed in the Lv and Bai, and *Csa3G904140* is control immature fruit color of cultivated cucumber [26].

### Functional analysis of DEGs

In order to understand the role of DEGs in the formation of fruit skin color, three categories were classified including biological process, molecular function, and cellular components using GO (gene ontology) standardized classification system, and total of 67 GO were significantly enriched. In biological processes category, 46 GO terms were significantly enriched in DEGs, such as thylakoid membrane organization, photosynthesis and chlorophyll biosynthetic process. In molecular function category, two GO categories, including pigment binding and chlorophyll binding were found to be enriched. In cellular component category, 19 GO terms, such as photosystem I, photosystem II, plastoglobule, chloroplast envelope, chloroplast, microtubule, chloroplast stroma and chloroplast thylakoid, were identified to enrich in DEGs (Table S4). Then, we used KEGG pathway database to examine the DEGs-associated pathways. The top 20 pathway enrichment of annotated DEGs across the comparisons of Lv and Bai was shown in Fig. 5b. Related genes of carbon mechanism, amino sugar and nucleotide sugar metabolism, photosynthesis, porphyrin chlorophyll metabolism and phenoylpropanoid biosynthesis were intensively enriched (Fig. 5b).

**Fig. 6:**
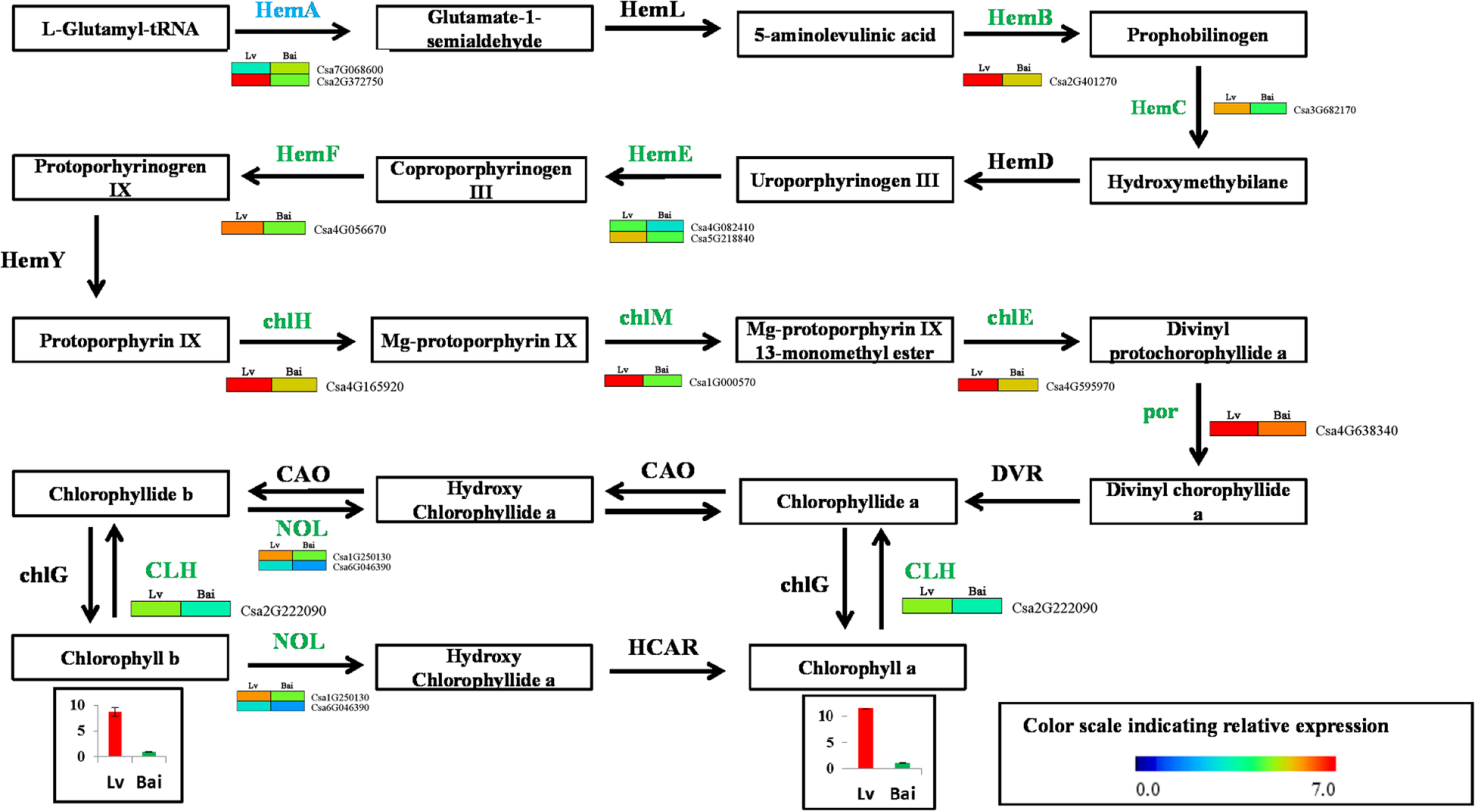
The detailed information on DEGs involved in the pathway of chlorophyll metabolism. HemA, glutamyl-tRNA reductase; HemL, glutamate-1-semialdehyde 2,1-aminomutase; HemB, porphobilinogen synthase; HemC, hydroxymethylbilane synthase; HemD, uroporphyrinogen-III synthase; HemE, uroporphyrinogen decarboxylase; HemF, coproporphyrinogen III oxidase; chlH, magnesium chelatase subunit H; chlM, magnesium-protoporphyrin O-methyltransferase; chlE, magnesium-protoporphyrin IX monomethyl ester; por, protochlorophyllide reductase; DAR, divinyl chlorophyllide a 8-vinyl-reductase; CAO, chlorophyllide a oxygenase; chlG, chlorophyll/bacteriochlorophyll a synthase; NOL, chlorophyll(ide) b reductase; HCAR, 7-hydroxymethyl chlorophyll a reductase; CLH, chlorophyllase

The GO and KEGG analysis results indicated that DEGs involved in chlorophyll metabolism-related pathway, these results are consist with chlorophyll a and chlorophyll b difference between Lv and Bai. Therefore, we further studied DEGs participate in chlorophyll metabolism in detail and established a predicted chlorophyll biosynthetic pathway (Fig. 6). Fourteen DEGs were identified in chlorophyll biosynthetic pathway. Interestingly, most these DEGs were down-regulated expression in Bai compared to Lv, except one DEG (*Csa7G068600*).

### Regulatory network of predicted flavonoid, and anthocyanidin biosynthetic pathways

**Fig. 7:**
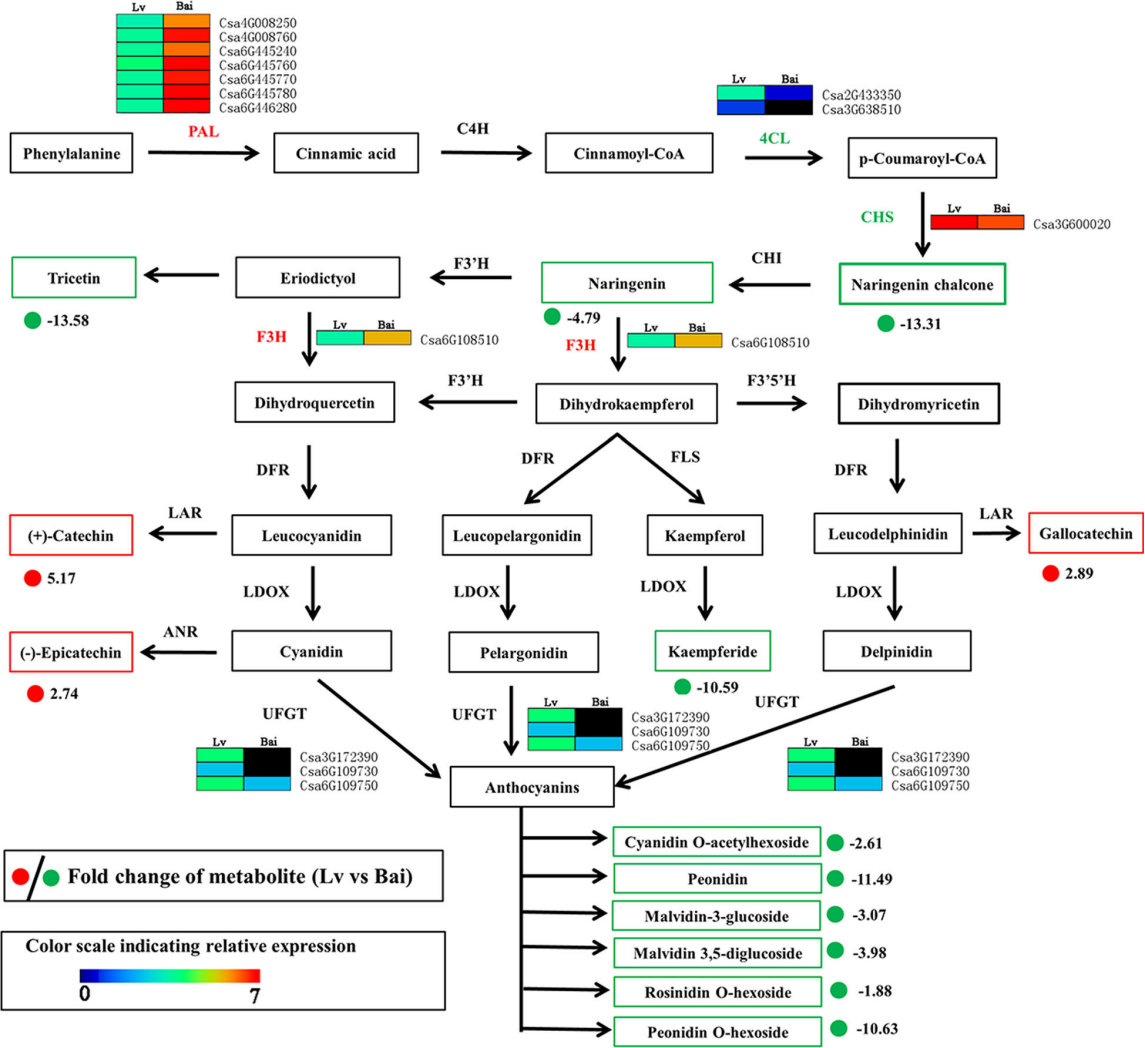
Regulatory network of predicted flavonoid biosynthesis in Lv and Bai. PAL, phenylalanine ammonia-lyase; C4H, trans-cinnamate 4-hydroxylase; 4CL, 4-coumarate: CoA ligase; CHS, chalcone synthase; CHI, chalcone isomerase; F3H, flavanone 3-hydroxylase; F3’H, flavonoid 3′-hydroxylase; DFR, dihydroflavonol 4-reductase; FLS, flavonol synthesis; LDOX, leucoanthocyanidin dioxygenase; UFGT, UDP glucose-flavonoid 3-*O*-glcosyl-transferase; LAR, leucocyanidin reductase; ANR, anthocyanidin reductase

In order to better understand the relationship between metabolites and genes in predicted flavonoid biosynthesis between Lv and Bai, the metabolites and gene were combined to establish a predicted network (Fig. 7). The 13 metabolites were significantly expressed difference between Lv and Bai, including 10 down-regulated metabolites (Naringenin chalcone, Naringenin, Tricetin, Kaemferide and six Anthocyanins (Cyanidin O-acetylhexoside, Peonidin, Malvidin 3-O-glucoside, Malvidin 3, 5- diglucoside, Peonidin O-hexoside, and Rosinidin O-hexoside)) and three up-regulated metabolites ((+)-Catechin,(−)-Epicatechin, Gallocatechin). Seven *PAL* genes (*Csa4G008250*, *Csa4G008760*, *Csa6G445240*, *Csa6G445760*, *Csa6G445770*, *Csa6G445780* and *Csa6G446280*) and one *F3H* (*Csa6G108510*) gene were up-regulated in the Bai compared to Lv. In addition, two structural genes *4CL* (*Csa2G433350* and *Csa3G638510*) were showed − 2.44- and − 1.70-fold decrement, and *CHS* (*Csa3G600020*) was − 1.14-fold down-regulation, this could largely explain the high accumulation of Naringenin chalcone and Naringenin in the Lv. Simultaneously, three *UFGT* genes (*Csa3G172390*, *Csa6G109730* and *Csa6G109750*) showed − 5.30-, − 5.86- and − 1.65fold down-regulation in the Bai, which also supports six anthocyanins significantly down-regulated in Bai compared with Lv.

## Discussion

Combining omics analysis of diverse genetic resources provides crucial information in understanding molecular basis of plant traits such as fig fruit color [22], Lilium “Tiny Padhye” bicolor development [23], peanut resistance on salt stress [27]. The cucumber shows a large variation in fruit skin colour, such as dark green, yellow, light green and milk white, these colours are characteristic of species or specific genotypes. In particular, the dark green and light green skin color cucumber cultivars have generated great interest in customer. In the study, we characterized two different cucumber on fruit skin color (Lv and Bai) using RNA-seq and metabolome. Lv exerted dark green with much more chlorophyll content and more closely arranged epidermal cells. Through analysis of different metabolites, flavones, flavanones, flavonols, and anthocyanins were mostly responsible for skin color differences. In addition, combining transcript level by RNA-seq, we found that several DEGs related to chlorophyll synthesis, anthocyanins synthesis and TFs were possibly involved in the color development.

### Regulatory network of DEGs associated with chlorophyll synthesis pathway for skin color in lv and Bai

Chlorophyll is an important pigment for determined the skin color of many fruits. Chlorophyll synthesis has been well studied and important related genes for chlorophyll synthesis have been found in leave and fruits [8, 28]. Gang et al. [29] found that *BpGLK1* the function for decreased chlorophyll content and defective chloroplast development by physiological and ultrastructural analysis. In addition, many key genes of coding enzymes were involved in chlorophyll synthesis pathway, such as *HemA*, *HemB*, *chlH*, *chlM*, *por*, *NOL* [3, 4]. For example, *HemA*, which is initiated enzyme for chlorophyll synthesis in plastid, catalyzes biosynthesis of 5-aminolevulinic acid from glutamyl-tRNA [30]. The *ChlH* catalyzes protoporphyrin IX to form Mg-protoporphyrin IX. The magnesium protoporphyrin IX monomethyl ester formation was catalyzed magnesium protoporphyrin IX in chlorophyll synthesis pathway by *ChlM* [31]. The *por* is an important enzyme that catalyzes protochlorophyllide to generate chlorophyllide, and this step is a critical intermediate step in converting chlorophyll [32]. Here, 14 DEGs were identified in chlorophyll synthesis pathway. The expression of DEGs in synthesis of chlorophylls synthesis pathway, including one *HemA*, one *HemB*, one *HemC*, two *HemE*, one *HemF*, one *chlH*, one c*hlM*, one *chlE*, one *por*, one *CLH*, two *NOL*, were down-regulated in Bai compared to Lv. These down-regulated expressions of many key genes involved in chlorophyll synthesis pathway may lead to inhibition of chlorophyll a and chlorophyll b synthesis. These results were consistent with higher accumulation of chlorophyll and more chloroplast in Lv than Bai.

### Analysis of anthocyanins and flavonols synthesis for fruit skin color

Metabolites are the final products of cell biological regulation process [33] and metabolomic analysis enables us investigate the relationship between biological processes and plant characteristic [34] . The content of anthocyanins and flavonoids has crucial effect on fruit color and taste [22, 35]. The metabolome data combining with transcriptome profiling were discovered genes involved in anthocyanins and flavonols synthesis, thus searching for useful information to illustrate phenomenon of different color in cucumber fruit. Anthocyanins are the final products of the flavonoid biosynthetic pathways, our search showed many DEGs are differently expressed between Lv and Bai in this pathway, such as upstream *4CL*, *CHS*, *F3H* and *UFGT*. Previous studies showed *4CL* genes play an essential role at the divergence point flavonols aynthesis [36]. The *CHS* has been found responsible for the anthocyanin biosynthesis during petal coloration in *Malus* crabapple [37]. Our study identified two *4CL* (*Csa2G433350* and *Csa3G638510*) and *CHS* (*Csa3G600020*) genes were down-regulated in Bai compared with Lv, and two metabolites (Naringenin chalcone and Naringenin) also down-regulated in Bai. It indicated that *CHS* was significantly repressed in Bai, and lead to down-regulation of two important metabolites in anthocyanin synthesis. In addition, we detected six types of anthocyanins have differently expressed between Bai and Lv. In anthocyanins biosynthesis, the glycosyl is a crucial progress, which catalyzed by *UFGT* in *Arabidopsis* [38]. The *UFGT* expression was associated with anthocyanin accumulation in different plant [39, 40]. Our results showed that three *UFGT* expressions are suppressed in Bai, it maybe explain six types of anthocyanins down-regulation in Bai compared to Lv. Other searcher found that the Cyanidin-3-O-rhamnoglucoside, one type of anthocyanins is main anthocyanin and played an important role in skin of figs [41, 42]., while cyanidin-3-O-rhamnoglucoside was not detected in our data, indicating it might be not main anthocyanin in cucumber fruit skin.

### Analysis of TFs involved in biosynthesis of anthocyanin in lv fruit skin

Anthocyanins and flavonoid synthesis are regulated by several structural genes and TFs such as MYB, bHLH and WDR proteins. The bHLH proteins can interact with R2R3-MYBs from various subgroups, and form ternary complexes with WDR. The MBW (MYB–bHLH–WDR) complexes participated in flavonols, anthocyanins, and proanthocyanidins (PAs) biosynthesis pathway [43–45]. Among these, MYB as major determinant element for anthocyanin accumulation regulation, could activate some pivotal anthocyanin biosynthetic genes by interacting with bHLH respectively [46, 47]. Ectopic overexpression of pear *PyMYB10* in *Arabidopsis* contributed to its pigmentation in immature seeds, indicating *PyMYB10* as positive factor in regulating anthocyanin accumulation [48]. Overexpression of peach *PpMYB10.1* in tobacco could increase the expression of *UFGT*, leading to higher anthocyanin accumulation and deeper red flowers in transgenic tobacco [49]. Similarly, MYB could regulate anthocyanin biosynthesis by regulating the expression of *UFGT* in grape [50] and apple [51]. In our research, 16 MYB TFs were detected by transcriptome, and expression levels of eight MYBs were up-regulated in fruit skin of Lv compared with Bai, indicating MYBs in Lv contributed the expression of related genes involved in anthocyanin synthesis.

The bHLH played an important role in anthocyanin synthesis by forming a complex with MYBs [41]. Overexpression of *SlPRE2*, an atypical bHLH, accelerated seedling morphogenesis and produced yellowing ripen fruits with reduced chlorophyll and carotenoid in tomato fruit [52]. Overexpressing *Arabidopsis GLABRA3* (bHLH) exhibited higher anthocyanin accumulation than control sample in tomato fruit [53]. In this study, 11 bHLHs were up-regulated in Lv fruit skin, while seven bHLHs were significantly down-regulated compared with Bai, suggesting bHLHs function as different roles in biosynthesis of anthocyanin.

## Conclusions

Overall, the regulation mechanism of fruit skin color on cucumber was firstly carried out by metabolome and RNA-Seq. The content of chlorophyll a, chlorophyll b and carotenoid were higher in Lv than Bai, and cytological observation showed more chloroplast existed in Lv. Crucial anthocyanins and flavonols responsible for fruit skin color development showed significantly different expression between two cucumber genotypes by metabolome. Several genes, especially *por* and *NOL*, *CHS* and *UFGT*, which play important roles in chlorophyll synthesis and anthocyanins biosynthesis pathway, respectively, were differently expressed between Bai and Lv fruit skin. Taken together, these different metabolites and genes identified in our study provide an important metabolic and functional role for chlorophyll synthesis and anthocyanins biosynthesis pathway in cucumber skin color.

## Methods

### Plant materials and growth conditions

Two cucumber high inbred lines (Lv and Bai) were used in this study, and were inbred line selected by our research group after multi-generation self-crossing. Lv and Bai were both South China type variety with contrasting differences in fruit skin color. Seeds were germinated on culture dish in a dark environment. Then, the seedlings were grown in a culture room under 14 h/10 h with 28 °C/18 °C in day/night. When plants were grown to two true leaf stages, and were transferred to the open field in Baiyun Area, Guangzhou City, China.

### Analysis of chlorophyll and carotenoid content in fruit skin between Bai and lv

Chlorophyll and carotenoid content of fruit skin from Lv and Bai were measured on the basis of the procedure described by Xie et al. (2019) [6]. Approximately, 0.2 g fruit skin were placed in 5 ml solution (9:1 = acetone: 0.1 M NH_4_OH).The samples were centrifuged at 3000 r for 20 min, and supernatants were collected. The same process was repeated thrice and the supernatants were collected using hexane. Finally, the mixed supernatant was measured by spectrophotometer at the absorption wavelengths of 663 nm and 645 nm (Beckman Coulter DU-800, CITY, USA). The measurements were performed with biological replicates.

### Scanning and transmission electron microscopy

After cucumber fruit skin was air-dried, the epidermis cells were visualized under a HITACHI SU8020 variable pressure SEM (Hitachi, Japan). For TEM assay, fruit skin were cut into small pieces, and were collected for fixation, and the process was performed as according to Wang et al. (2019) [54].

### Metabolomic analysis

Metabolite profiling was performed using a widely targeted metabolome method by Wuhan Metware Biotechnology Co., Ltd. (Wuhan, China) (http://www.metware.cn/). Freezing-dried fruit skin was crushed into powder using a mixer mill (MM 400, Retsch). The fruit skin (1 cm wide and 0.2 cm thick along the fruit lengthwise) were sampled 10–15 days after female flowers open, and three replicates each of Lv and Bai. A total of 100 mg powder was extracted overnight at 4 °C with 1.0 ml 70% aqueous methanol, then centrifuged at 10, 000 g for 10 min. After that, these extracts were absorbed, filtrated, and analyzed by an LC-ESI-MS/MS system. Analytical conditions were based on the procedures as described in Wang et al. (2017) [22]. Quantification of metabolites was carried out using a MRM method [33]. Metabolites with significant differences in content were set with thresholds of variable importance in projection (VIP) ≥1 and fold change ≥2 or ≤ 0.5 [55].

### Transcriptome analysis

The fruit skin (1 cm wide and 0.2 cm thick along the fruit skin lengthwise in the middle part) were sampled 10–15 days after female flowers open. A total of twelve samples (three replicates each of Lv and Bai) were prepared for RNA extraction based on the instruction of TRIZOL reagent (TaKaRa, Japan). RNA was purified and concentrated using an RNeasy MinElute clean up kit (Qiagen, Germany) after RNA extraction. Then, about 2.5 μg RNA from each sample was prepared for constructing sequencing libraries and the library quality was detected by Agilent Bioanalyzer 2100 system. The library preparations were sequenced on Illumina Hiseq2500 platform and 125/150 bp paired-end reads were generated. Index of the reference genome was built using Bowtie v2.2.3 and paired-end clean reads were aligned to the reference genome using TopHat v2.0.12 [56].

Gene expression level was analysis by FPKM (fragments per kilobase per million reads) method [57]. The FPKM of genes were calculated by Cuffquant and cuffnorm (v2.2.1) (v2.2.1) [58]. DESeq2 was used to identify DEGs according to the two criteria(fold change ≥2 or ≤ 0.5and q ≤ 0.01). WEGO software and KEGG database were employed to GO enrichment and bigological pathway enrichment, respectively [59, 60].

### Quantitative real-time PCR (qRT-PCR) validation

The qRT-PCR reaction was performed on ABI PRISM 7900HT machine (Applied Biosystems, USA) by using the SYBR Premix Ex Taq Kit (TaKaRa, Japan), and qRT-PCR reaction process was performed according to Wang et al. (2019) [54]. All primers used in qRT-PCR were listed in Table S5.

## Supplementary information

**Additional file 1: Figure S1.** Single cell on average between Lv and Bai.

**Additional file 2: Figure S2.** The correlation analysis and principal component analysis (PCA) in Lv and Bai fruit.

**Additional file 3: Figure S3.** The number of DEGs belonging to different transcription factor families detected in Lv and Bai.

**Additional file 4: Figure S4.** Relative expression of genes related to transcriptional factors. Data is presented as the mean ± standard deviation (*n* = 9).

**Additional file 5: Table S1.** Differentially metabolites in the skin of Lv and Bai fruit.

**Additional file 6: Table S2.** Overview of reads from Lv and Bai fruit skin by RNA-seq.

**Additional file 7: Table S3.** Differentially expressed genes between Lv and Bai fruit skin.

**Additional file 8: Table S4.** GO enrichment analysis for DEG in Lv and Bai fruit skin.

**Additional file 9: Table S5.** List of primer of qRT-PCR.

## Data Availability

All the sequencing data were submitted to NCBI Sequence Read Archive database under accession number PRJNA647135.

## Abbreviations

qRT-PCR: Quantitative real-Time PCR
DEG: Differentially expressed genes
TEM: Transmission electron microscopy
SEM: Scanning electron microscope
TIC: Total ions current
MRM: Multiple reactions monitoring
KEGG: Kyoto Encyclopedia of Genes and Genomes
PCA: Principal component analysis
TF: Transcription factor
HemA: Glutamyl-tRNA reductase
HemL: Glutamate-1-semialdehyde 2,1-aminomutase
HemB: Porphobilinogen synthase
HemC: Hydroxymethylbilane synthase
HemD: Uroporphyrinogen-III synthase
HemE: Uroporphyrinogen decarboxylase
HemF: Coproporphyrinogen III oxidase
chlH: Magnesium chelatase subunit H
chlM: Magnesium-protoporphyrin O-methyltransferase
chlE: Magnesium-protoporphyrin IX monomethyl ester
por: Protochlorophyllide reductase
DAR: Divinyl chlorophyllide a 8-vinyl-reductase
CAO: Chlorophyllide a oxygenase
chlG: Chlorophyll/bacteriochlorophyll a synthase
NOL: Chlorophyll(ide) b reductase
HCAR: 7-hydroxymethyl chlorophyll a reductase
CLH: Chlorophyllase
PAL: Phenylalanine ammonia-lyase
C4H: Trans-cinnamate 4-hydroxylase
4CL: 4-coumarate: CoA ligase
CHS: Chalcone synthase
CHI: Chalcone isomerase
F3H: Flavanone 3-hydroxylase
F3’H: Flavonoid 3′-hydroxylase
DFR: Dihydroflavonol 4-reductase
FLS: Flavonol synthesis
LDOX: Leucoanthocyanidin dioxygenase
UFGT: UDP glucose-flavonoid 3-O-glcosyl-transferase
LAR: Leucocyanidin reductase
ANR: Anthocyanidin reductase
