# Supplementary figures and images for: Metabolome and transcriptome analyses reveal chlorophyll and anthocyanin metabolism pathway associated with cucumber fruit skin color
Source: BMC Plant Biol. 2020 Aug 24;20:386. doi: 10.1186/s12870-020-02597-9 (PMC7444041; doi:10.1186/s12870-020-02597-9)

## Slide 1
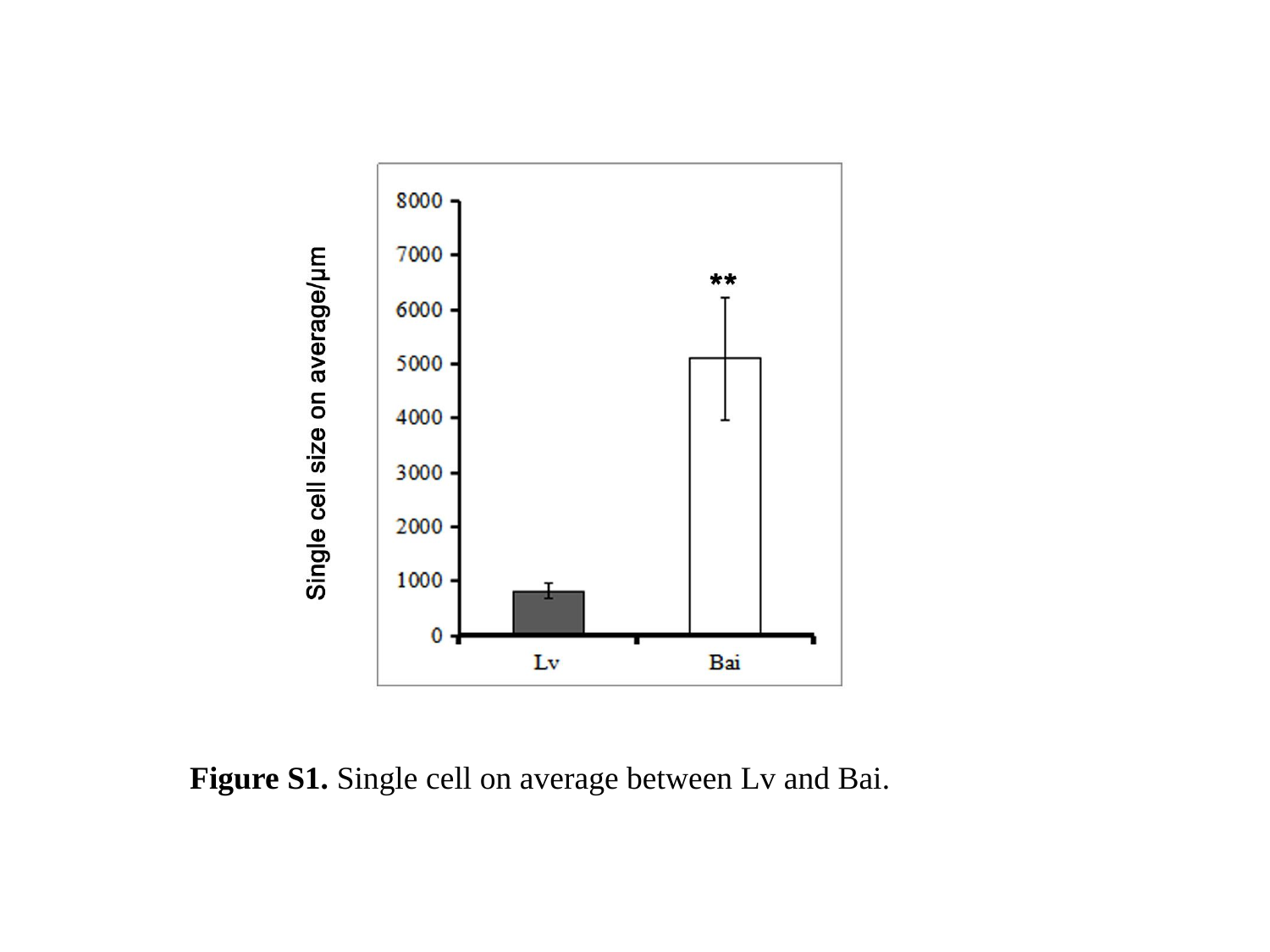

Figure S1. Single cell on average between Lv and Bai.

Supplement: Supplementary file 1 — Additional file 1: Figure S1. Single cell on average between Lv and Bai. [file 12870_2020_2597_MOESM1_ESM.pptx]
