# Supplementary material for: Metabolome and transcriptome analyses reveal chlorophyll and anthocyanin metabolism pathway associated with cucumber fruit skin color
Source: BMC Plant Biol. 2020 Aug 24;20:386. doi: 10.1186/s12870-020-02597-9 (PMC7444041; doi:10.1186/s12870-020-02597-9)
Supplement: Supplementary file 2 — Additional file 2: Figure S2. The correlation analysis and principal component analysis (PCA) in Lv and Bai fruit. [file 12870_2020_2597_MOESM2_ESM.pptx]

## Slide 1
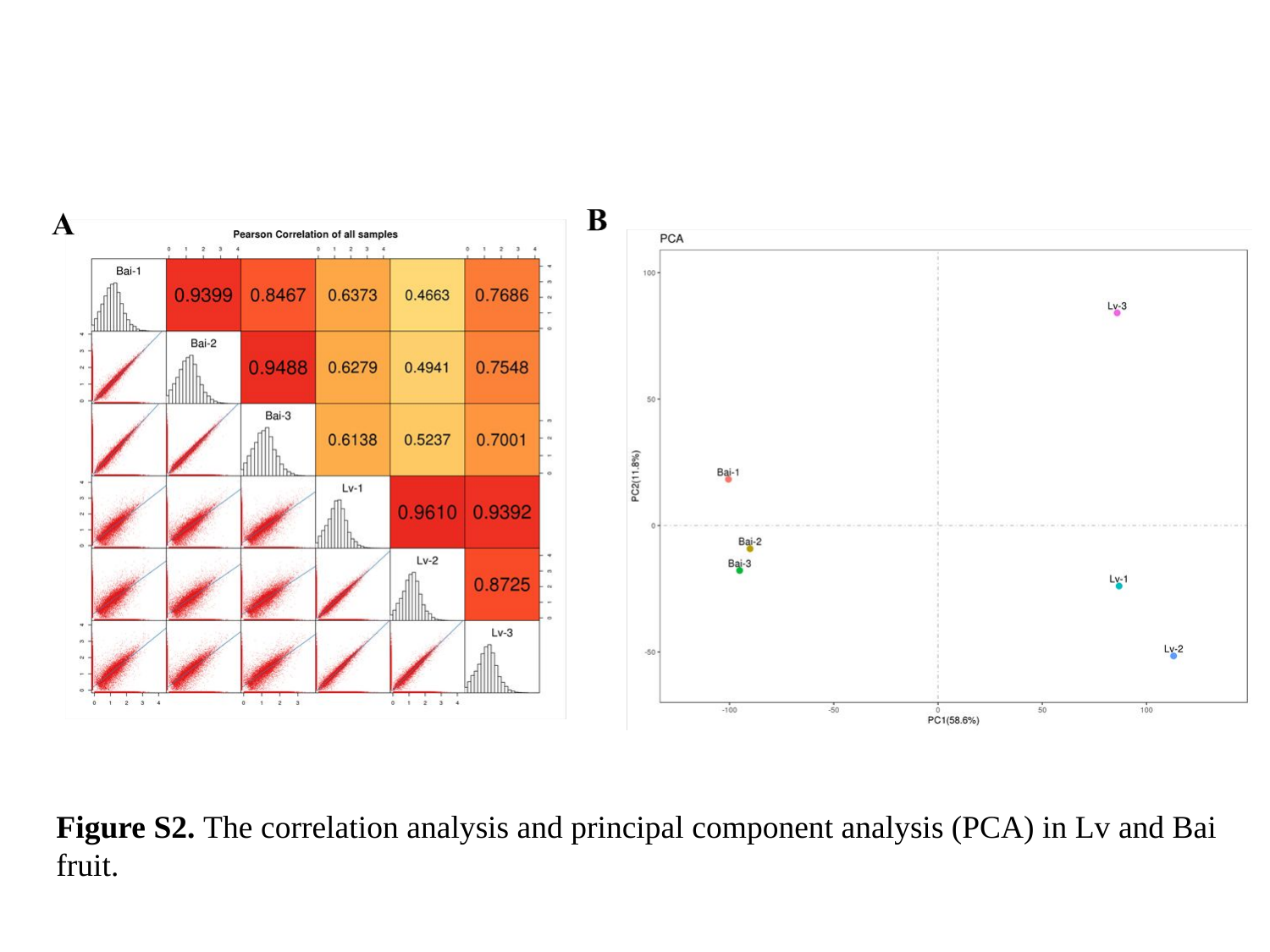

Figure S2. The correlation analysis and principal component analysis (PCA) in Lv and Bai fruit.
