# Supplementary material for: Metabolome and transcriptome analyses reveal chlorophyll and anthocyanin metabolism pathway associated with cucumber fruit skin color
Source: BMC Plant Biol. 2020 Aug 24;20:386. doi: 10.1186/s12870-020-02597-9 (PMC7444041; doi:10.1186/s12870-020-02597-9)
Supplement: Supplementary file 3 — Additional file 3: Figure S3. The number of DEGs belonging to different transcription factor families detected in Lv and Bai. [file 12870_2020_2597_MOESM3_ESM.pptx]

## Slide 1
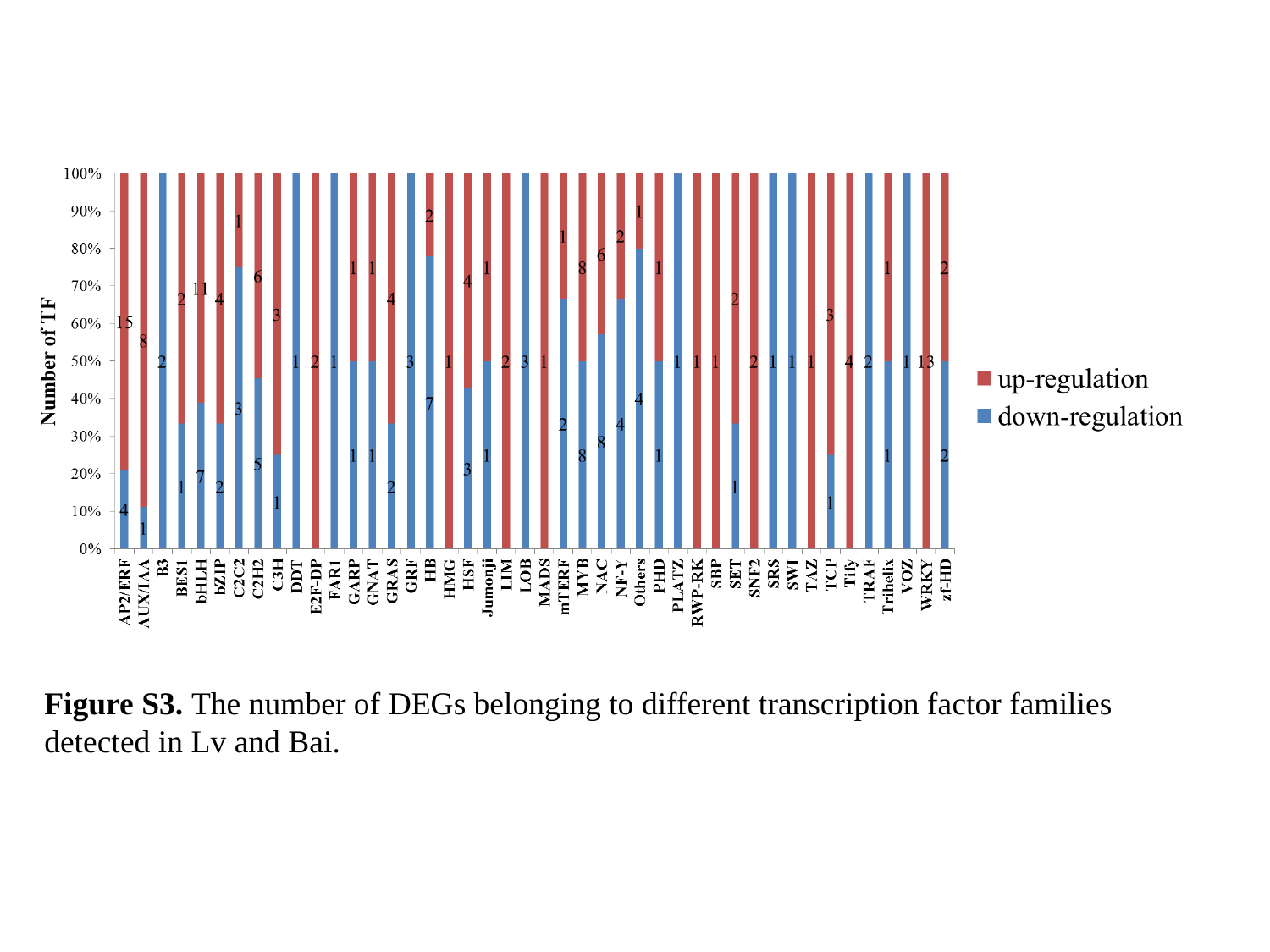

Figure S3. The number of DEGs belonging to different transcription factor families detected in Lv and Bai.
