# Supplementary material for: Metabolome and transcriptome analyses reveal chlorophyll and anthocyanin metabolism pathway associated with cucumber fruit skin color
Source: BMC Plant Biol. 2020 Aug 24;20:386. doi: 10.1186/s12870-020-02597-9 (PMC7444041; doi:10.1186/s12870-020-02597-9)
Supplement: Supplementary file 4 — Additional file 4: Figure S4. Relative expression of genes related to transcriptional factors. Data is presented as the mean ± standard deviation (n = 9). [file 12870_2020_2597_MOESM4_ESM.pptx]

## Slide 1
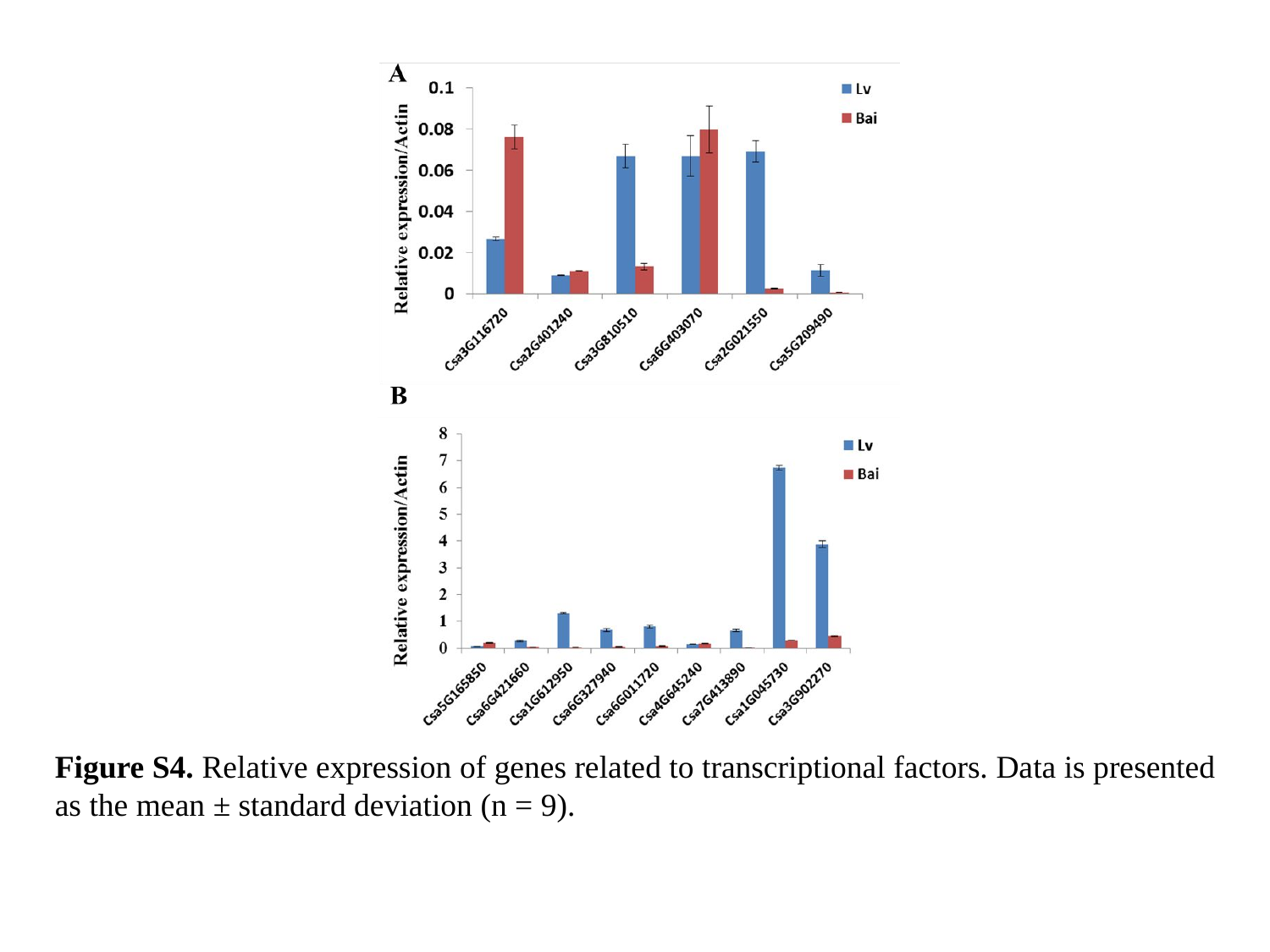

Figure S4. Relative expression of genes related to transcriptional factors. Data is presented as the mean ± standard deviation (n = 9).
